# Pharmacokinetics and Pharmacodynamics of Florfenicol in Plasma and Synovial Fluid of Pigs at a Dose of 30 mg/kg_bw_ Following Intramuscular Administration

**DOI:** 10.3390/antibiotics12040758

**Published:** 2023-04-14

**Authors:** Zoltán Somogyi, Patrik Mag, Réka Simon, Ádám Kerek, Pál Szabó, Ervin Albert, Imre Biksi, Ákos Jerzsele

**Affiliations:** 1 Department of Pharmacology and Toxicology, University of Veterinary Medicine, 1078 Budapest, Hungary; 2 National Laboratory of Infectious Animal Diseases, Antimicrobial Resistance, Veterinary Public Health and Food Chain Safety, University of Veterinary Medicine, 1078 Budapest, Hungary; 3 Research Center for Natural Sciences, Center for Structural Study, MS Metabolomics Laboratory, 1117 Budapest, Hungary; 4 Department of Pathology, University of Veterinary Medicine Budapest, 2225 Üllő, Hungary; 5 SCG Diagnostics Ltd., 2437 Délegyháza, Hungary

**Keywords:** florfenicol, pharmacokinetic, MIC, AUC, AUC_24h_/MIC, synovial fluid, pig

## Abstract

A major problem of our time is the ever-increasing resistance to antimicrobial agents in bacterial populations. One of the most effective ways to prevent these problems is to target antibacterial therapies for specific diseases. In this study, we investigated the in vitro effectiveness of florfenicol against *S. suis*, which can cause severe arthritis and septicemia in swine herds. The pharmacokinetic and pharmacodynamic properties of florfenicol in porcine plasma and synovial fluid were determined. After a single intramuscular administration of florfenicol at 30 mg/kg_bw_, the AUC_0–∞_ was 164.45 ± 34.18 µg/mL × h and the maximum plasma concentration was 8.15 ± 3.11 µg/mL, which was reached in 1.40 ± 0.66 h, whereas, in the synovial fluid, these values were 64.57 ± 30.37 µg/mL × h, 4.51 ± 1.16 µg/mL and 1.75 ± 1.16 h, respectively. Based on the MIC values of the 73 *S. suis* isolates tested, the MIC_50_ and MIC_90_ values were 2 µg/mL and 8 µg/mL, respectively. We successfully implemented a killing–time curve in pig synovial fluid as a matrix. Based on our findings, the PK/PD breakpoints of the bacteriostatic (E = 0), bactericidal (E = −3) and eradication (E = −4) effects of florfenicol were determined and MIC thresholds were calculated, which are the guiding indicators for the treatment of these diseases. The AUC_24h_/MIC values for bacteriostatic, bactericidal and eradication effects were 22.22 h, 76.88 h and 141.74 h, respectively, in synovial fluid, and 22.42 h, 86.49 h and 161.76 h, respectively, in plasma. The critical MIC values of florfenicol against *S. suis* regarding bacteriostatic, bactericidal and eradication effects in pig synovial fluid were 2.91 ± 1.37 µg/mL, 0.84 ± 0.39 µg/mL and 0.46 ± 0.21 µg/mL, respectively. These values provide a basis for further studies on the use of florfenicol. Furthermore, our research highlights the importance of investigating the pharmacokinetic properties of antibacterial agents at the site of infection and the pharmacodynamic properties of these agents against different bacteria in different media.

## 1. Introduction

Antimicrobial resistance (AMR) is one of the leading health issues of our time, in human and veterinary medicine alike. This is underlined by the increasingly stringent regulation of the use and consumption of antibacterial agents [1]. In addition to the time-consuming and costly development of new antibacterial agents, the other solution is the repositioning of already authorized agents for new, different indications. In this case, a good method may be to use pharmacokinetic/pharmacodynamic (PK/PD) analysis [2]. Florfenicol is an example of why the accurate dose and time interval of the treatment for different indications should be determined, as it has been shown to promote the selection of florfenicol-resistant *Escherichia coli* strains in the microbiota despite high concentrations in the pig gastrointestinal tract [3].

Florfenicol, a member of the phenicol group, is a broad-spectrum antibacterial agent with a bacteriostatic mode of action, which is achieved by binding to the 50S subunit of the ribosome via inhibition of the enzyme peptidyl transferase [4,5,6,7,8]. It is widely used in the pig industry to treat respiratory diseases caused by *Pasteurella multocida*, *Actinobacillus pleuropneumoniae*, *Mycoplasma hyopneumoniae*, *M. hyorhinis*, Glässer-disease caused by *Glaesserella parasuis* and septicemia, polyserositis, meningitis and arthritis caused by *Streptococcus suis*. Furthermore, it is also used to treat bovine, sheep, goats, poultry and fish [5,9,10,11,12,13,14,15,16,17,18,19,20,21,22,23]. Although most products containing florfenicol are not authorized for diseases caused by *S. suis* in the European Union, studies to date have demonstrated its efficacy in septicemia caused by *S. suis* [23,24,25].

In order to provide data for the use of florfenicol in additional diseases caused by *S. suis*, the pharmacokinetic (PK) properties of florfenicol at the site of infection need to be investigated [26,27]. The pharmacokinetics of florfenicol in pigs have already been investigated in plasma and the lung. The pharmacokinetic properties of florfenicol vary widely between individual pigs [28]. Overall, it has excellent absorption and distribution in the pig body system, with a very low binding to plasma protein [24,28,29,30,31,32]. In a previous study, we investigated the pharmacokinetics of florfenicol in pig synovial fluid at a dose of 15 mg/kg_bw_ following a single intramuscular administration. Based on this, it was concluded that florfenicol can only be used to treat arthritis caused by *S. suis* in pig if the minimum inhibitory concentration (MIC) of *S. suis* is less than or equal to 1.42 µg/mL [23]. In addition to the pharmacokinetic parameters, it is very important to continuously monitor the sensitivity of *S. suis* strains against florfenicol [26] because most studies show that the susceptibility of *S. suis* to florfenicol is not always as clear, as indicated by the MIC_50_ and MIC_90_ values of 2 µg/mL or lower in almost all countries, which is the breakpoint set by Clinical and Laboratory Standards Institute [15,16,22,23,24,25].

Similar studies needed to use PK/PD analysis to determine the dose and duration of florfenicol treatments and the time interval between two drug administrations as accurately as possible.

In a previous study, we investigated the PK of florefnicol in pig plasma and synovial fluid at a dose of 15 mg/kg_bw_ following a single intramuscular administration, in which the results were inconclusive and we could not conclude that florfenicol can be recommended for the treatment of pig arthritis caused by *S. suis* in the approved dosage regimen. The aim of the present study was to determine the plasma and synovial PK of florfenicol at a dose of 30 mg/kg_bw_ following a single intramuscular injection; thus, at a dose higher than the approved dosage regimen. In addition, the in vitro efficacy of florfenicol against *S. suis* bacteria isolated from clinical lesions in Hungary was investigated using PK/PD analysis. Moreover, our aim was to test the efficacy of florfenicol against *S. suis* strains in an in vitro experiment, characterized, in this case, by synovial fluid. For this purpose, we prepared killing curves of an *S. suis* isolate (SS96) in pig synovial fluid, plasma and cation-adjusted Mueller–Hinton broth (CA-MHB).

## 2. Results

### 2.1. Pharmacokinetics of Florfenicol

**Figure 1:**
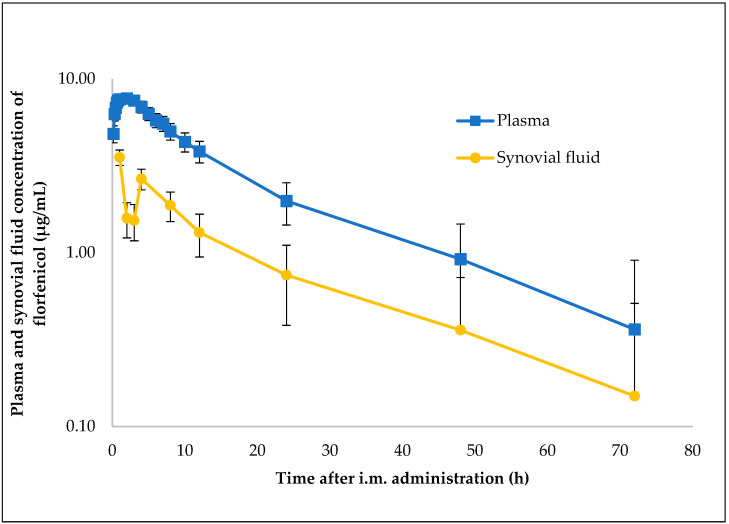
Semi-logarithmic plot illustrating the concentration–time curve of florfenicol in plasma and synovial fluid samples of pigs after a single i.m. administration of 30 mg/kg_bw_ (n = 8).

Pharmacokinetic parameters were computed via non-compartmental analysis from plasma and synovial fluid concentration data for 8 pigs. Florfenicol was administered intramuscularly at a single injection of 30 mg/kg_bw_. Table 1 presents mean PK parameters and standard deviation. The semi-logarithmic plasma and synovial fluid concentration–time curves of florfenicol after single i.m. administration of 30 mg/kg_bw_ are illustrated in Figure 1. The mean C_max_ of 8.15 ± 3.11 µg/mL in plasma was achieved with a T_max_ of 1.40 ± 0.66 h. Florfenicol reached peak concentration in the synovial fluid more slowly after i.m. administration, and the C_max_ 4.51 ± 1.16 µg/mL was achieved in 1.75 ± 1.16 h. The mean plasma and synovial fluid AUC_24h_ following i.m. administration of florfenicol were 102.91 ± 19.90 µg/mL × h and 41.90 ± 16.93 µg/mL × h, respectively.

### 2.2. MIC of Florfenicol against S. suis

**Figure 2:**
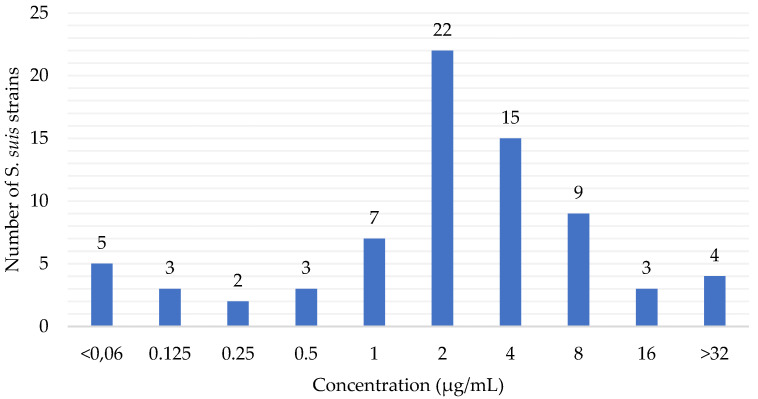
Minimum inhibitory concentration (MIC) distribution of florfenicol against *S. suis* in Hungary between 2018 and 2022 (n = 73).

The florfenicol susceptibility of 73 *S. suis* isolates from pigs is summarized in Figure 2. The MIC_50_ and MIC_90_ were determined from the MIC values of 73 *S. suis* clinical isolates against florfenicol. According to the EUCAST [33] (The European Committee on Antimicrobial Susceptibility Testing) ECOFF (epidemiological cut-off) value (≤4 µg/L), 78.08% (57) of the *S. suis* isolates were wild-type, whereas 21.92% (16) were considered as non-wild-type to florfenicol. The CLSI breakpoints, however, indicate that 57.53% (42) of *S. suis* isolates are susceptible, 20.55% (15) are intermediate and 21.92% (16) are resistant to florfenicol.

### 2.3. PK and PD of Florfenicol after i.m. Administration of 30 mg/kg_bw_ to Eight Healthy Pigs

The AUC/MIC_50_ and AUC/MIC_90_ values were calculated based on the AUC_24h_ values determined in the plasma (102.91 ± 19.90 µg/mL × h) and synovial fluid (41.90 ± 16.93 µg/mL × h) of eight pigs for 24 h and the MIC_50_ and MIC_90_ values (2 µg/mL, 8 µg/mL) of 73 *S. suis* isolates. The AUC/MIC_50_ and AUC/MIC_90_ values in pig plasma were 51.45 ± 9.95 h and 12.86 ± 2.49 h, respectively. The AUC/MIC_50_ and AUC/MIC_90_ values in pig synovial fluid were 20.95 ± 8.47 h and 5.24 ± 2.12 h, respectively.

### 2.4. In Vitro Killing–Time Curves of Florfenicol against S. suis 96 Strain in Three Different Media (Pig Synovial Fluid, Pig Plasma, CA-MHB)

**Figure 3:**
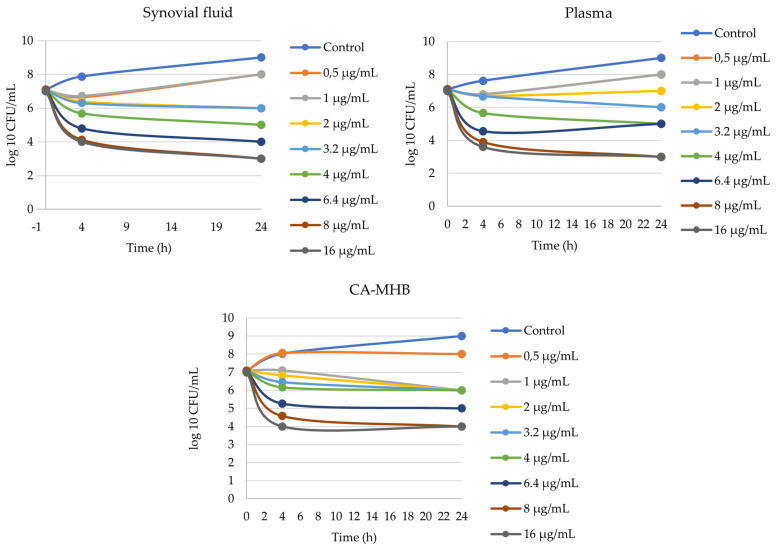
In vitro killing–time curves of florfenicol against S. suis 96 strain in three different media (synovial fluid, plasma, CA-MHB).

The MIC values of the investigated SS96 *S. suis* strain against florfenicol were 2 µg/mL, 2 µg/mL and 2 µg/mL in pig synovial fluid, pig plasma and CA-MHB, respectively. There was no difference between MIC values in different media. In all three media, a 3 log_10_ bacterial count decrease was achieved after 4 h at concentrations of 8 µg/mL and 16 µg/mL of florfenicol. The bacterial count reduction was 1 log_10_ or more when the concentration of florfenicol was greater than 2 µg/mL. The in vitro killing–time curves of florfenicol against SS96 *S. suis* strains in three different media are shown in Figure 3.

### 2.5. PK/PD Integration

**Figure 4:**
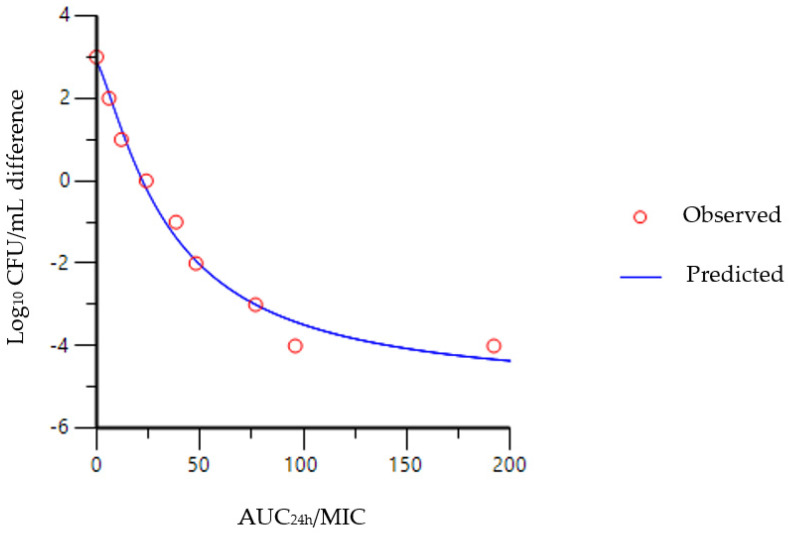
Sigmoidal Emax correlation between SS96 bacterial count (CFU/mL) and in vitro AUC_24h_/MIC of florfenicol, illustrating the values required for bacteriostatic, bactericidal and eradication effects in synovial fluid in pig.

The relationship between synovial fluid AUC_24h_/MIC and the reduction in bacterial counts is shown in Figure 4. The AUC_24h_/MIC values required to result in a bacteriostatic effect were 22.22 h, 22.42 h and 14.21 h for florfenicol in synovial fluid, plasma and CA-MHB, respectively, as shown in Table 2. The corresponding values for bactericidal (E = −3) activity were 76.88 h, 86.49 h and 163.16 h, respectively. AUC_24h_/MIC values for bacterial eradication (E = −4) were higher in synovial fluid and plasma at 141.74 h and 161.76 h, respectively, whereas, in CA-MHB, florfenicol did not reach this level even at the highest concentration (16 µg/mL).

### 2.6. Critical MIC Values of Florfenicol against S. suis Bacteriostatic, Bactericidal and Eradication Effects

Dividing the AUC_24hss_ values calculated for eight pigs by the AUC_24h_/MIC breakpoints ratios gives the concentrations as MIC values, which can result in bacteriostatic, bactericidal and eradication effects in synovial fluid and plasma. Numerically, they were 2.91 ± 1.37, 0.84 ± 0.39 and 0.46 ± 0.21 for *S. suis* in synovial fluid and 7.34 ± 1.52, 1.90 ± 0.40 and 1.02 ± 0.21 in plasma, respectively (Table 3).

## 3. Discussion

Florfenicol is characterized by a high lipid solubility and low protein binding, the latter being less than 15% in pig plasma, which results in a high V_d_ value for florfenicol [34,35]. The pharmacokinetics of florfenicol have been extensively studied in pigs; however, apart from our previous study, the concentration of florfenicol in synovial fluid has not yet been determined [23]. Our results regarding plasma florfenicol data are similar to previous studies. The maximum plasma concentration of florfenicol administered intramuscularly at a dose of 15 mg/kg_bw_ was 3.04 ± 1.82 µg/mL, reached in 1.94 ± 0.87 h, which is almost identical to the results of our previous study, where C_max_ and T_max_ were 3.58 ± 1.51 µg/mL and 1.64 ± 1.74 h, respectively, in porcine plasma, whereas, in synovial fluid, they were 2.73 ± 1.2 µg/mL and 3.4 ± 1.67 h, respectively [23,32]. Following the intramuscular administration of florfenicol at a dose of 20 mg/kg_bw_, the maximum plasma concentration was 7.3 ± 6.0 µg/mL, which was reached in 2.3 ± 1.2 h [31]. In the present study, florfenicol was administered intramuscularly at a dose of 30 mg/kg_bw_, after which the C_max_ and T_max_ were 8.15 ± 3.11 µg/mL, which was reached in 1.40 ± 0.66 h. Here, the C_max_ is higher than in a similar study, where the C_max_ after application of the same dose was 4.44 ± 1.02 µg/mL [24]. All of this suggests that florfenicol is rapidly absorbed from the site of administration and rapidly distributed throughout the pig body system, including the synovial fluid. It can be clearly seen that higher values are also obtained in the synovial fluid following the administration of higher doses. Following administration at a dose of 30 mg/kg_bw_, the C_max_, AUC_0–∞_ and T_max_ were 4.51 ± 1.16 µg/mL, 64.57 µg/mL × h and 1.75 ± 1.16 h, respectively. Figure 1 shows that lower concentrations are measured in the synovial fluid after the intramuscular administration of florfenicol compared to the first and fourth hours, which can be explained by the flip-flop kinetics of florfenicol, as the vehicle delays the absorption of the drug after intramuscular and subcutaneous administration.

Regarding the area under the concentration–time curve, florfenicol reaches higher values in plasma than in the synovial fluid, as already described in our previous publication; however, here, it is also valid at a higher dose. Since it has been shown in horses that, in acute arthritis, greater drug concentrations are achieved in synovial fluid than in healthy joints, we can assume that this is also the case in the synovial fluid of pigs [36].

In the present study, MIC values of 73 *S. suis* isolates were determined and the MIC_50_ and MIC_90_ were calculated. Among these values, the MIC_50_ is the same as the MIC_50_ value determined in a previous study, whereas the MIC_90_-values are closer to the results of studies conducted in other countries based on the results of our present study [16,37,38]. Although, in an Italian study, only 3% of 78 *S. suis* strains were resistant based on the CLSI breakpoint, in our study, 16% of 73 *S. suis* strains were resistant to florfenicol [39]. The susceptibility of *S. suis* to antibacterial agents is influenced by many factors, so continuous monitoring is recommended, even on a farm-by-farm basis, which is mandatory under current legislation, as antibacterial therapy can be used based on prior antibiotic susceptibility testing.

In the present study, we were the first to grow *S. suis* bacteria in pig synovial fluid and to implement an in vitro killing–time curve, the results of which will help to refine treatment protocols for arthritis caused by *S. suis* strains with florfenicol. The matrix effect, supported by several studies, could not be demonstrated in this study, as the MIC value of SS96 (2 µg/mL) was the same in all three media (synovial fluid, plasma, CA-MHB). The difference between the three media was observed in the failure to achieve the 4 log_10_ bacterial count reduction in the CA-MHB, even with the highest concentration of florfenicol (16 µg/mL). As no killing–time curve has been performed in synovial fluid before, our data could not be compared with other studies. Our data in plasma and in CA-MHB differ from the results of a previous study in which the AUC_24h_/MIC values for the bacteriostatic (E = 0), bactericidal (E = −3) and eradication (E = −4) effects of florfenicol determined ex vivo were 37.89 ± 4. 25 h, 44.02 ± 4.85 h and 46.42 ± 6.45 h in pig serum [24]. We obtained lower values for the bacteriostatic effect in all three media, whereas higher values were obtained for the bactericidal and eradication effects. The reasons for the difference may be that the two studies did not use the same *S. suis* strains, or that Lei at al. [24] used tryptic soy broth whereas we used CA-MHB. The differences in plasma could be due to the presence of antibodies. The importance of these studies is to characterize the behavior of the bacteria in the medium, with which in vitro models can be built at the site of infection. Modeling the site of infection will provide a basis for refining the use of antibiotics and thus increasing the effectiveness of treatments [40].

Based on the results of our present study, a bacteriostatic effect of florfenicol in swine arthritis caused by *S. suis* can be achieved below MIC values of 2 µg/mL, whereas, for septicemia, an MIC value of 7 µg/mL is recommended as a threshold value. A bactericidal effect can be expected if the MIC value for *S. suis* strains in arthritis is ≤0.8 µg/mL or in septicemia is ≤1.9 µg/mL in plasma. The critical MIC values for the eradication effect in arthritis and septicemia are ≤0.46 µg/mL and ≤1 µg/mL, respectively. It is important to note that these thresholds do not apply after intramuscular administration at the authorized dose of 15 mg/kg_bw_, but after a single intramuscular administration at the dose of 30 mg/kg_bw_ used in our study, in which case, as we are deviating from the instructions for use, it is now the responsibility of the veterinarian to determine the withdrawal period, which should be taken into account for all therapies. On the basis of the known MIC values, our previous studies and other studies [23,24], we believe that florfenicol has a place in the treatment of swine arthritis caused by S. suis, but it would be worthwhile to perform further pharmacokinetic studies in an infection model and to confirm the results of the studies performed so far with clinical trials.

## 4. Materials and Methods

### 4.1. Experimental Animals and Design

In this study, we used 8 male pigs (Danish Landrace × Danish Yorkshire × Danish Duroc) with an average body weight (BW) of 28.93 ± 3.64 kg. In pig herds, clinical cases are most common between 4–8 weeks of age [41]. In our study, piglets were selected at 11 weeks of age, as this is also the age at which S. suis arthritis is most likely to occur, as maternal antibodies are certainly depleted. The animals were purchased from a local commercial pig farm in Hungary. The animals had not received any antimicrobial treatment prior to the experiment and were vaccinated against porcine circovirus 2 at 4 weeks of age. They were kept at 22–23 °C with adequate ventilation conditions, the relative humidity was 70% and the number of hours of light and darkness was 12 h each. Standard commercial feed and drinking water were provided ad libitum without medication prior to the experiment and no medication other than florfenicol was given to the pigs during the experiment. The pigs arrived at the experimental place a week earlier and did not show any clinical signs during this time, so it can be said that the investigation was carried out on clinically health animals. The study was authorized by the Local Animal Welfare Committee of the University of Veterinary Medicine, Budapest, and by the Government Office of Pest County, Food Chain Safety, Plant Protection and Soil Conservation Directorate, Budapest, Hungary (admission No. PE/EA/00367-6/2022).

Florfenicol (Nuflor injection A.U.V., Intervet International B.V., Boxmeer, Netherlands) was administered intramuscularly at a dose rate of 30 mg/kg_bw_. The drug was administered to the left neck muscles of the pigs after blind samples were taken. Subsequently, blood samples were then taken at the following times: 10, 20, 30, 40, 50, 60 min, 2, 3, 4, 5, 6, 7, 8, 10, 12, 24, 48 and 72 h, while synovial fluid samples were taken at the following time points: 1, 2, 3, 4, 8, 12, 24, 48 and 72 h. Blood samples were collected from the cranial vena cava of the animals using a 21 G × 2” needle and lithium heparin blood tube, and the blood samples were centrifuged at 1482× *g* for 10 min after sampling. For synovial fluid sampling, joint puncture was performed in the carpal and tarsal joints in continuous rotation using a 22 G × 1 ½” needle and 1 mL syringe. Samples were stored in low binding tubes at −80 °C until analysis.

### 4.2. Tandem Mass Spectrometry Analysis

Florfenicol was quantitated on the basis of the method published earlier [23]. Briefly: a Sciex 6500QTrap tandem mass spectrometer (Sciex, Framingham, MS, USA) was used in multiple reaction monitoring (MRM) mode, where the quantifier and qualifier transitions were 358.2/241 and 358.2/170, respectively. The mass spectrometer was operated in electrospray ionization with spray voltage of 5000 V. An Agilent 1100 HPLC system was coupled to the MS. A Kinetex XB C18 (50_2.1 mm, 2.6_m) column (Phenomenex) was applied for the separation by using water and acetonitrile, both containing 0.1% formic acid, in gradient mode. Five-point calibration model was applied. Analyst 1.6.3 software was used for data processing and controlling the measurements.

### 4.3. Pharmacokinetic Analysis

A non-compartmental pharmacokinetic analysis was used to determine the pharmacokinetic parameters of florfenicol in plasma and synovial fluid. The maximum drug concentration (C_max_) and the time of onset of maximum drug concentration (T_max_) were computed. The area under the 24 h concentration–time curve (AUC_24h_) and the area under the infinity extrapolated curve (AUC_0–∞_) were determined using a linear trapezoidal method. The half-life (T_½_), total body clearance (Cl/F) and mean residence time (MRT_∞_) were computed. For florfenicol, binding to plasma protein in pig was below 5% as it is negligible, so binding to plasma proteins was not considered [34,35,42]. Pharmacokinetic calculations and statistical analysis were performed using the Phoenix WinNonLin 8.3 software (Certara, Princeton, NJ, USA).

### 4.4. Minimum Inhibitory Concentration

The antibiotic susceptibility to florfenicol of 73 *S. suis* isolates from clinical samples of pig origin in Hungary were determined by broth microdilution method according to the CLSI (Clinical and Laboratory Standards Institute) description [43]. The isolation of *S. suis* was performed in 2022. The isolates were collected from Hungarian pig farms, each isolated from clinical lesions of dissected pigs. In each case, the samples were taken from untreated pigs.

The broth microdilution method was performed using CA-MHB (Mueller–Hinton Broth 2, Merck KGaA, Darmstadt, Germany). The isolates were stored at −80 °C and incubated in the presence of 5.0% CO_2_ at 37 °C for 24 h as recommended before broth microdilution method. After incubation, for the determination of the germ count, the bacterial suspensions were centrifuged at 3000× *g* for 10 min, washed in sterile physiological saline (Salsol solution infusion, TEVA Gyógyszergyár Zrt., Debrecen, Hungary), centrifuged again at 3000× *g* for 10 min and finally resuspended in physiological saline. The optical density of the suspensions at 600 nm was set to 0.1 (OD_600_ = 0.1), with the appropriate amount of physiological saline, which corresponded to 10^8^ colony forming units (CFU)/mL bacterial density and a standard of 0.5 on the MacFarland scale. A suspension of 5 × 10^5^ CFU/mL was prepared with a 200-fold dilution. The germ count of the suspensions was tested with inoculation to blood agar plates and counting the number of CFUs. The sensitivity of *S. suis* isolates to florfenicol was tested by the broth microdilution method in the range of 32 µg/mL to 0.0625 µg/mL. Each isolate was included in the investigation with positive and negative control wells. This was followed by incubation period, and then the MIC values were read. This was the lowest concentration where no bacterial growth was detected. MIC_50_ and MIC_90_ values were computed as the MIC that inhibited the growth of 50% and 90%, respectively, of the isolates in different clusters. Determination of MIC and calculation of MIC_50_ and MIC_90_ were performed according to CLSI.

### 4.5. PK and PD of Florfenicol

PK/PD indices, calculated individual 8 pigs’ PK values of florfenicol after i.m. administration of 30 mg/kg_bw_ and MIC_50_- and MIC_90_-values of 73 *S. suis* isolates were: AUC_24h_/MIC_50_, AUC_24h_/MIC_90_. Among the PK/PD relationships, AUC/MIC was selected as it better describes the clinical efficacy of long-acting formulations [44,45]. Since, in veterinary medicine, the AUC/MIC is not always projected to 24 h, we consider that, in this case, it is appropriate to use the unit h to denote this value [2].

### 4.6. In Vitro Model of Pig Synovial Fluid

To investigate the efficacy of florfenicol, an in vitro pig synovial fluid model was established and implemented as follows. Synovial fluid samples for bacterial growth were collected within 24 h prior to experiments from clinically healthy and untreated pigs. During the sampling, the skin surface of the joints was properly prepared, i.e., disinfected after shaving the hair, and then sampled for synovial fluid as described above. The syringes were then transported cooled at 4 °C and the needles were removed from syringes under laboratory conditions under laminar box to avoid contamination of the synovial fluid. Subsequently, 10 µL of each sample was inoculated on blood agar plate (Bak-Teszt Kft., Budapest, Hungary) and incubated at 37 °C for 18–24 h to check the sterility of the synovial fluids. Under the laminar box, the samples were individually placed in sterile tubes and stored at 4 °C until the start of the experiment. In the case where the synoval fluid was frozen (−20 °C), we observed a strong gelation, rendering the synovial fluids unsuitable for further investigation.

Before starting the experiment, we checked the blood agar results and included only those synovial fluid samples that were sterile; then these samples were in the ratio of 9:1 with sterile physiological saline, with the exception of the control (0 µg/mL florfenicol) sample, in order to inject the appropriate amount of florfenicol to form a two-fold dilution (0.125, 0.25, 0.5, 1.0, 2.0, 4.0, 8.0, 16.0 µg/mL) and to facilitate handling of the synovial fluid. These solutions were used to set up the killing–time curve.

### 4.7. Killing–Time Curve In Vitro

In order to generate the killing–time curves, we used an isolate of *S. suis* SS96, which was isolated from pig arthritis. Its MIC was determined in both pig serum and pig synovial fluid. The growth of the SS96 isolate was tested in florfenicol-free CA-MHB, pig serum and pig synovial fluid, which were the controls in the study, and the efficacy of florfenicol was also tested in the same media at the following concentrations: 0.125, 0.25, 0.5, 1.0, 2.0, 4.0, 8.0 and 16.0 µg/mL. Prior to the test, SS96 isolate was incubated in CA-MHB for 18 h in ambient air at 37 °C to achieve the appropriate initial germ count. This was determined as described above and the starting germ count was adjusted to 6.5 × 10^4^ CFU/mL in CA-MHB, pig serum and pig synovial fluid media. Subsequently, the different media were incubated in ambient air at 37 °C for 24 h at different concentrations of florfenicol. Following incubation, ten-fold dilution series were prepared and inoculated onto blood agar and, after incubation, in ambient air at 37 °C for 24 h with 5% CO_2_, the 24-h germ count was determined.

### 4.8. PK/PD Integration and Breakpoints

The sigmoidal E_max_ equation was used to model AUC_24h_/MIC data from killing–time curves using the non-linear regression program WinNonLin to predict plots of log_10_ change in CFU/mL versus AUC_24h_/MIC. PK/PD breakpoints were determined for three levels of growth inhibition after 24 h incubation: E = 0, bacteriostatic, which is a 0 log_10_ reduction in CFU/mL; E = −3, bactericidal, 3 log_10_ reduction in CFU/mL; and E = −4, 4 log_10_ reduction in bacterial count [2].

Dividing the AUC_24hss_ values calculated for 8 pigs in vivo by the AUC_24h_/MIC breakpoints ratios gives the concentrations as critical MIC values, which can result in bacteriostatic, bactericidal and 4 log_10_ number reductions in synovial fluid and plasma in vitro.

$$E=E_{0}-\frac{E_{\max}*C_{\left(t\right)}^{\mathrm{Gamma}}}{{\mathrm{EC}}_{50}^{\mathrm{Gamma}}+C_{\left(t\right)}^{\mathrm{Gamma}}}$$

Hill equation. E = log_10_-based change in live cell count, E_0_ = initial log_10_-based bacterial live cell count, E_max_ = maximum (response) kill capacity, C(t) = AUC_24h_/MIC, EC_50_ = in vitro concentration of florfenicol capable of half the maximum kill capacity Gamma= Hill coefficient (slope of the curve).

## Figures and Tables

**Table 1:** Plasma and synovial fluid PK parameters (mean ± SD) of florfenicol (Nuflor) in pigs following intramuscular administration of 30 mg/kg_bw_ (n = 8).

PK Parameters	Units	Plasma	Synovial Fluid
C_max_	µg/mL	8.15 ± 3.11	4.51 ± 1.16
T_max_	h	1.40 ± 0.66	1.75 ± 1.16
T_1/2_	h	18.19 ± 11.40	12.27 ± 7.45
AUC_24h_	µg/mL × h	102.91 ± 19.90	41.90 ± 16.93
AUC_0–∞_	µg/mL × h	164.45 ± 34.18	64.57 ± 30.37
Cl/F	L/h/kg	0.19 ± 0.04	0.59 ± 0.32
MRT_0–∞_	h	25.56 ± 15.53	21.16 ± 12.21

C_max_: maximum plasma and synovial fluid concentrations; T_max_: time to peak plasma and synovial fluid concentrations; T_1/2_: terminal elimination half-life; AUC_24h_: area under the curve for 24 h; AUC_0–∞_: area under the curve from zero time to infinity; AUC_24hss_: area under the curve in steady-state conditions over 24 h; Cl/F: drug clearance scaled by bioavailability; MRT_0–∞_: mean residence time.

**Table 2:** PK/PD breakpoints determined from the sigmoidal E_max_ inhibition equation in porcine synovial Fluid, plasma and CA-MHB.

Parameters	Units	Synovial Fluid	Plasma	CA-MHB
Log E_max_	CFU/mL	−8.00 ± 1.00	−6.76 ± 1.71	−6.89 ± 2.63
Log EC_50_	h	34.99 ± 7.10	46.80 ± 16.94	45.28 ± 47.16
Log E_0_	CFU/mL	2.87 ± 0.36	1.77 ± 0.50	2.02 ± 0.43
Gamma	-	1.28 ± 0.27	1.41 ± 0.58	0.76 ± 0.33
AUC_24h_/MIC for bacteriostatic effect (E = 0)	h	22.22	22.42	14.21
AUC_24h_/MIC for bactericidal effect (E = −3)	h	76.88	86.49	163.16
AUC_24h_/MIC for 4 log_10_ reduction (E = −4)	h	141.74	161.76	- *

Emax = maximum (response) killing capacity, EC50 = in vitro concentration of florfenicol capable of half the maximum killing capacity expressed in hours, E0 = initial bacterial viable cell count, Gamma = Hill coefficient (slope of the curve).* Florfenicol did not reach this level even at the highest concentration (16 µg/mL).

**Table 3:** Critical MIC values of florfenicol against S. suis regarding bacteriostatic, bactericidal and eradication effects in pig synovial fluid and plasma.

Effect	Unit	Synovial Fluid	Plasma
Bacteriostatic	µg/mL	≤2.91 ± 1.37	≤7.34 ± 1.52
Bactericidal	µg/mL	≤0.84 ± 0.39	≤1.90 ± 0.40
Eradication	µg/mL	≤0.46 ± 0.21	≤1.02 ± 0.21

## Data Availability

The data presented in this study are available on request from the corresponding author.
